# Cholesterol Efflux: Does It Contribute to Aortic Stiffening?

**DOI:** 10.3390/jcdd5020023

**Published:** 2018-05-01

**Authors:** Shutan Liao, Craig S. McLachlan

**Affiliations:** 1 Rural Clinical School, Faculty of Medicine, University of New South Wales, Sydney, NSW 2052, Australia; shutan.liao@unsw.edu.au; 2 The First Affiliated Hospital of Sun Yat-sen University, Guangzhou 510080, China

**Keywords:** aortic stiffness, cholesterol efflux, reverse cholesterol transport, ATP-binding cassette transporters

## Abstract

Aortic stiffness during cardiac contraction is defined by the rigidity of the aorta and the elastic resistance to deformation. Recent studies suggest that aortic stiffness may be associated with changes in cholesterol efflux in endothelial cells. This alteration in cholesterol efflux may directly affect endothelial function, extracellular matrix composition, and vascular smooth muscle cell function and behavior. These pathological changes favor an aortic stiffness phenotype. Among all of the proteins participating in the cholesterol efflux process, ATP binding cassette transporter A1 (ABCA1) appears to be the main contributor to arterial stiffness changes in terms of structural and cellular function. ABCA1 is also associated with vascular inflammation mediators implicated in aortic stiffness. The goal of this mini review is to provide a conceptual hypothesis of the recent advancements in the understanding of ABCA1 in cholesterol efflux and its role and association in the development of aortic stiffness, with a particular emphasis on the potential mechanisms and pathways involved.

## 1. Introduction

Arterial stiffness develops from a complex interaction between stable and dynamic changes involving structural and cellular elements of the vessel wall. Aortic stiffness is a consequence of pathophysiological alterations involving endothelial cells, vascular smooth muscle cells (VSMCs), extracellular matrix (ECM), inflammatory responses, and other functional elements [1]. The aorta not only serves as a conduit for blood to reach peripheral tissues, but also regulates systolic blood pressure and maintains diastolic blood pressure by its elastic properties (Windkessel effect) [2]. Normal compliance and contractility of the aorta help in damping the fluctuation in blood pressure over the cardiac cycle and assist in the maintenance of organ perfusion during diastole. Vessel compliance is considered to be related to the composition of the ECM—particularly collagens and elastin [3]. VSMCs provide an active tension component within the aorta contributing to its contractility properties [4]. The endothelium also modulates aortic stiffness due to its capacity to modulate smooth muscle tone. Dedifferentiated VSMCs synthesize large amounts of ECM proteins and matrix-modifying enzymes and are therefore proposed to be a significant regulator of ECM remodeling and arterial stiffness [5,6].

A stiffened aorta, as a consequence of fractures of the elastic lamina, replacement of elastin by collagens, local inflammation, VSMC infiltration, necrosis, and calcification, opposes systolic distention. When this occurs, hemodynamic factors require a greater amount of force to accommodate the stroke volume, which leads to an increase in systolic blood pressure and a decrease in diastolic blood pressure, and therefore high pulse pressure. The increase in systolic blood pressure and velocity can result in an increase in left ventricular afterload and increased sheer stress to vessels. Meanwhile, a decrease in diastolic blood pressure causes decreased perfusion in both the peripheral and coronary circulations. It is well recognized that aortic stiffness precedes future systemic hypertension and amplifies the pathogenesis of hypertension by changing the pressure oscillations and pulsatile shear stress throughout the cardiovascular system [7,8,9]. Additionally, aortic stiffness is believed to be the earliest detectable manifestations of adverse structural and functional changes within the aortic wall [10]. Aortic stiffness is recognized as an independent predictor of left ventricular diastolic dysfunction and heart failure with preserved ejection fraction [11,12,13], as well as cardiovascular events and mortality [14,15].

Aortic stiffness, as well as high-density lipoprotein (HDL), particularly its activity in cholesterol efflux, are associated with cardiovascular risk factors such as hypertension [16], metabolic syndrome [17,18], and coronary artery disease [19]. Cholesterol efflux affects endothelial cells, ECM composition, and VSMC function and behavior [20,21], suggesting an intimacy between arterial stiffness and the cholesterol efflux in cardiovascular diseases. 

## 2. Potential Mechanisms of Aortic Stiffness and Cholesterol Efflux

Increasing evidence supports the potential role of cholesterol efflux in aortic stiffness. Apolipoprotein (Apo) A1 is the major protein component of HDL and is essential for HDL biogenesis and function. Recent studies report that HDL-3 cholesterol and Apo B/Apo A1 ratio are associated with arterial stiffness [22,23]. Oxidized low-density lipoprotein (LDL) exhibits potentially atherogenic actions, including the transformation of macrophages to foam cells. Previous reports demonstrate that oxidized LDL decreases cholesterol efflux from endothelial cell membranes, which in turn leads to endothelial cell stiffening, changes in cellular force generation, and network formation [24,25]. These factors contribute to the arterial stiffness.

The cholesterol efflux pathway is thought to also play a key role in HDL anti-atherogenic pathways [26,27]. Indeed, the capacity of cholesterol efflux has been shown to be inversely associated with intima-media thickness [28]. However, this relationship is independent of HDL cholesterol levels, suggesting that cholesterol efflux may contribute to the process of aortic stiffening via other possible mechanisms.

**Figure 1:**
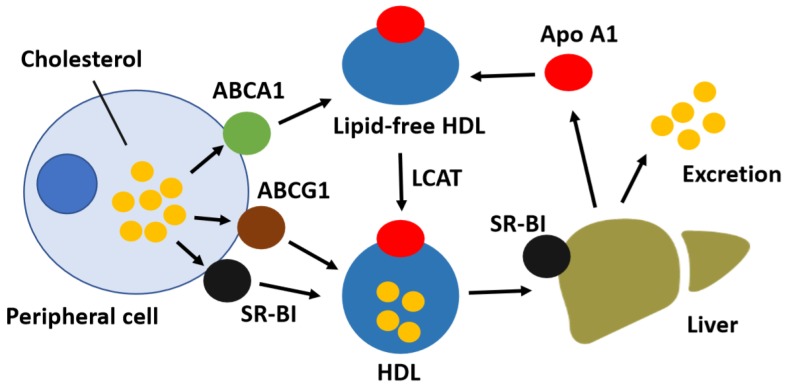
Schematic diagram of reverse cholesterol transport. Lipid-poor Apolipoprotein (Apo) A1 acquires free cholesterol from peripheral cells via ATP binding cassette transporter (ABC) A1, whereas the ABCG1 and scavenger receptor BI (SR-BI) transporters facilitate cholesterol efflux to high-density lipoprotein (HDL) particles. Free cholesterol is esterified to cholesteryl esters within nascent HDL particles by lecithin-cholesterol acyltransferase (LCAT), thereby generating mature HDL. The liver selectively takes up HDL-associated cholesteryl esters via SR-BI and excretes HDL-derived cholesterol into the bile as free cholesterol or as bile acids after conversion.

Reverse cholesterol transport is a complex multistep process. The initial and critical step, by which free cholesterol is exported from peripheral cells and subsequently transferred to HDL particles, is known as cholesterol efflux [29]. Increased cholesterol efflux prevents subsequent cardiac events by initiating the removal of excessive peripheral cholesterol for transport to the liver and subsequent removal [30]. Multiple proteins affect the rate and capacity of cholesterol efflux, including membrane transporters, mitochondrial enzymes, and Apo E [31,32]. In addition, enzymes involved in lipid storage and oxidation pathways affect the cholesterol efflux capacity by changing the intracellular cholesterol pool size. Extracellular lipid acceptors such as Apo A1 or HDL also impact the rate at which free cholesterol and phospholipids are transported from the cells [33]. Three cholesterol transporters, namely the ATP binding cassette transporter (ABC) A1, ABCG1, and scavenger receptor BI (SR-BI), are best characterized as primary mediators of cholesterol efflux (Figure 1) [34]. ABCA1 facilitates the efflux of free cholesterol to lipid-free Apo A1 in the plasma [35]; ABCG1 and SR-BI mediate free cholesterol efflux to mature HDL [36].

Transgenic animals with endothelial-specific overexpression of either ABCA1 or ABCG1 have been found to have enhanced cholesterol efflux, attenuated endothelial dysfunction, and reduced diet-induced aortic lesions [37,38]. Both ABCG1 and SR-BI are associated with endothelial nitric oxide synthase (eNOS) activity and endothelium-dependent vasorelaxation [39,40]. Interestingly, specific chemical inhibition of these transporters did not alter cholesterol efflux in cultured human aortic endothelial cells [41]. However, increased expressions of ABCA1 and ABCG1 were observed in aortic endothelial cells under shear stress [42], thus suggesting the participation of these transporters in reverse cholesterol transport only under simulated in vivo conditions. Another possibility is that other membrane or plasma proteins may also facilitate the cholesterol transporters in enhancing cholesterol efflux. Moreover, reduced function of ABCA1 might not only lead to cholesterol deposition in macrophages, but also contribute the dysfunctional changes of other cell types and inflammation in the arterial wall [43]. ABCA1 has been implicated in promoting the engulfment of apoptotic cells [44] and the release of prostaglandin into endothelial cells [45], resulting in anti-stiffening and anti-atherogenic effects through vasodilation as well as the inhibition of the expression of adhesion molecules, platelet aggregation, and monocyte adhesion [46]. Both ABCA1 and ABCG1 play beneficial roles in suppressing the oxidative burst and preserving the viability of macrophages following exposure to oxidized phospholipids [47].

In addition, a series of clinical trials report that angiotensin and fibroblast growth factor (FGF)-21 are associated with arterial stiffness. These tissue factors increase VSMC contraction, growth, and proliferation, as well as promoting cytoskeletal structural changes [48,49,50]. On the other hand, animal studies reveal that FGF-21 deletion aggravates diabetes-induced aortic remodeling and inflammation in type 1 diabetic mice [51] and Apo E(−/−) mice [52]; the blockage of endogenous angiotensin remarkably enhances contents of lipids and macrophages and decreases contents of VSMCs and collagens in aortic lesions in Apo E(−/−) mice [53]. As suggested by studies showing that angiotensin and FGF-21 enhance cholesterol efflux by upregulating ABCA1 and/or ABCG1 in macrophages [54,55], angiotensin and FGF-21 could act as compensatory mechanisms against arterial stiffness by enhancing cholesterol efflux. A proposed hypothesis is that ABCA1 and/or ABCG1-dependant cholesterol efflux may serve as mediators of arterial stiffness. However, further studies are warranted to elucidate this hypothesis.

The participation of cholesterol transporters in transient aortic stiffening is implicated by the application of intralipids. Intralipid is a fat emulsion of predominantly unsaturated fatty acids (UFAs) for intravenous administration. A number of studies suggest a close relationship between UFAs and cholesterol efflux [56,57,58,59]. Dose-dependent effects of UFAs on inhibiting cholesterol efflux from mouse macrophages by downregulating ABCA1 mRNA [56] and/or increasing degradation of ABCA1 protein [57,59] have been observed in in vitro studies. Indeed, the overexpression of either Apo A1 or ABCA1 can result in an increase in cholesterol efflux and a decrease in fatty acid synthesis [60]. Moreover, high-rate infusions of intralipid increase systemic vascular resistance as well as reduce vascular compliance in both humans and animals [61,62,63]. These data raise the possibility that cholesterol efflux may contribute to aortic stiffness via its interaction with fatty acid metabolism. However, other mechanisms, such as the suppression of eNOS activity and NO production by fatty acids, may be involved in vessel stiffening [64,65]. The effects of cholesterol efflux on arterial stiffness are further supported by a study revealing that cyclooxygenase-2 (COX-2) inhibition reduces arterial stiffness in hypertensive animals. This is achieved by diminishing vascular collagen deposition, normalizing altered elastin structure, and decreasing connective tissue growth factor gene expression [66]. An inhibitory effect of Apo A1 on COX-2 expression has also been observed [67,68]. Cholesterol efflux plays a role in the protection of aortic stiffness development by enhancing endothelial function, as well as inhibiting VSMC proliferation and inflammatory response.

Interestingly, changes of hemodynamic features related to or caused by aortic stiffness may also have an impact on functional cholesterol efflux. Recently, reduced cholesterol efflux capacity was observed in a minipig model of non-ischemic heart failure sustained by high-rate left ventricle pacing [69]. In hypertensive patients, the levels of cholesterol transporters were negatively associated with mean blood pressure, and the reduction of these transporters could be reversed by anti-hypertensive therapy [70]. Peroxisome proliferator-activating receptor gamma (PPARγ)-liver X receptor (LXR)-ABCA1 pathways are one of the key pathways in cholesterol efflux in macrophages [71]. It has been reported that PPARγ expression decreases in the lung vascular tissue in patients with pulmonary hypertension. It is likely that high fluid shear stress decreases PPARγ expression in vitro [72], and potentially impairs cholesterol efflux from macrophages. Studies in aortic endothelial cells subjected to laminar shear flow conditions demonstrate the upregulation of LXRα expression and the induction of transporter genes ABCA1 and ABCG1 [42,69].

## 3. The Role of ABCA1 in Pulse Wave Velocity

As the major cell-surface transporter in HDL-mediated cholesterol efflux, ABCA1 has been demonstrated to be associated with pulse wave velocity (PWV) in clinical studies. PWV is a frequently used surrogate marker of aortic stiffness and has been proposed to predict the risk of future cardiovascular disease events and total mortality [73]. ABCA1 gene mutation carriers are characterized by increased intima-media thickness [74,75], while ABCA1-dependent cholesterol efflux is inversely correlated with PWV in healthy subjects [76].

## 4. The Role of ABCA1 in Influencing Cellular Phenotypes

The importance of regulatory pathways in ABCA1 expression is critical in the regulation of VSMC differentiation, that is, a switch from a VSMC phenotype to a contractile synthetic form [77,78]. Apo A1 functions as the primary protein component of HDL and regulates the levels of ABCA1 by reducing the rate of ABCA1 protein degradation [79]. A marked variability in Apo A1 binding and Apo A1-mediated cholesterol efflux has been observed across different VSMC phenotypes, which exhibit different levels of contractility and rates of synthesis of ECM components including collagens, elastin, proteoglycans, cadherins, and integrins that regulate the stiffness of vessels [5,80,81]. Apo E upregulates ABCA1 expression [82], and is associated with the VSMC proliferation and the expressions of aortic stiffness-dependent ECM genes, such as collagen-I, fibronectin, and lysyl oxidase [83,84]. Recently, Castiglioni et al. demonstrated that HDL-3 counteracts VSMC phenotypic changes, increases VSMC markers, and decreases inflammation-associated markers after cholesterol-loading if a functional ABCA1 transporter is present [85]. A potential role of ABCA1 in aortic stiffness is suggested by its involvement in the regulation of VSMCs.

## 5. ABCA1 as an Anti-Inflammatory Receptor

Recent studies suggest that ABCA1 can function as an anti-inflammatory receptor to suppress the expression of inflammatory factors. ABCA1-deficient mice have increased inflammatory cell infiltration in the vessel wall and blood circulation [86]. ABCA1-mutation carriers exhibit a pro-inflammatory state as indicated by elevated levels of circulating inflammatory cytokines [87]. Interleukin (IL)-1β secretion is impaired by inhibitors of ABCA1 [88,89], while the interaction of Apo A1 with ABCA1-expressing macrophages suppresses the induction of the inflammatory cytokines IL-1β, IL-6, and tumor necrosis factor (TNF)-α [90]. IL-1β is reported to be responsible for the secretion and activation of matrix metalloproteinases (MMPs) 2 and 9 [91,92], while TNF-α inhibition improves endothelial function and reduces aortic stiffness [93,94]. MMPs are a family of over 20 enzymes that are capable of modifying all components of the ECM. MMPs participate in tissue development, remodeling, and repair, as well as atherosclerosis [95]. Of note, MMPs 2 and 9 are proposed to be the key elastolytic enzymes in ECM degradation [96]. It is reported that ABCA1 gene polymorphisms are associated with dyslipidemia and the production of some inflammatory cytokines including IL-6 and C-reactive protein (CRP) [97]. Moreover, one report suggests that the transcription levels of ABCA1 in Chinese populations are negatively associated with plasma CRP [98]. It is known that CRP promotes vascular inflammation and is found to be associated with aortic stiffness [99,100]. Furthermore, the nuclear receptors LXR and PPARγ, which are the key regulators of ABCA1 expression, have been documented to repress the inflammatory activity of MMP 2 and MMP 9 [101,102,103,104,105]. Therefore, it is hypothesized that ABCA1 may inhibit aortic stiffness by its anti-inflammatory properties.

## 6. Conclusions

There are several factors that are mechanistic in arterial stiffness related to cholesterol transport, such as endothelial dysfunction and inflammation. Clinical conditions such as hypertension, diabetes, dyslipidemia, and insulin resistance can contribute to bidirectional processes involved in the initiation and progression of arterial stiffening. Cholesterol efflux is associated with a number of structural and cellular changes that are associated with aortic stiffness. Hence, both the direct and indirect effects of cholesterol efflux are likely significant in understanding aortic stiffness development and pathophysiology.
